# A distinct microbiota composition is associated with protection from food allergy in an oral mouse immunization model

**DOI:** 10.1016/j.clim.2016.10.009

**Published:** 2016-10-24

**Authors:** Susanne C. Diesner, Cornelia Bergmayr, Barbara Pfitzner, Vera Assmann, Durga Krishnamurthy, Philipp Starkl, David Endesfelder, Michael Rothballer, Gerhard Welzl, Thomas Rattei, Thomas Eiwegger, Zsolt Szépfalusi, Heinz Fehrenbach, Erika Jensen-Jarolim, Anton Hartmann, Isabella Pali-Schöll, Eva Untersmayr

**Affiliations:** a Department of Pathophysiology and Allergy Research, Center of Pathophysiology, Infectiology and Immunology, Medical University of Vienna, Vienna, Austria; b Department of Pediatrics and Adolescent Medicine, Medical University of Vienna, Vienna, Austria; c Helmholtz Zentrum München, German Research Center for Environmental Health (GmbH), Department of Environmental Sciences, Research Unit Microbe-Plant Interactions, Research Group Molecular Microbial Ecology, Neuherberg, Germany; d Helmholtz Zentrum München, German Research Center for Environmental Health (GmbH), Scientific Computing Research Unit, Neuherberg, Germany; e Helmholtz Zentrum München, German Research Center for Environmental Health (GmbH), Department of Environmental Sciences, Research Unit Environmental Genomics, Neuherberg, Germany; f University of Vienna, Division of Computational Systems Biology, Vienna, Austria; g Division of Experimental Pneumology, Priority Area Asthma & Allergy, Research Center Borstel, Airway Research Center North (ARCN), Member of the German Center for Lung Research (DZL), Borstel, Germany; h Comparative Medicine, Messerli Research Institute of the Veterinary University of Vienna, Medical University of Vienna, University of Vienna, Vienna, Austria

**Keywords:** Food allergy, Allergen uptake, Intestinal barrier function, Cytokines, Microbiota, Bacterial community composition

## Abstract

In our mouse model, gastric acid-suppression is associated with antigen-specific IgE and anaphylaxis development. We repeatedly observed non-responder animals protected from food allergy. Here, we aimed to analyse reasons for this protection. Ten out of 64 mice, subjected to oral ovalbumin (OVA) immunizations under gastric acid-suppression, were non-responders without OVA-specific IgE or IgG1 elevation, indicating protection from allergy. In these non-responders, allergen challenges confirmed reduced antigen uptake and lack of anaphylactic symptoms, while in allergic mice high levels of mouse mast-cell protease-1 and a body temperature reduction, indicative for anaphylaxis, were determined. Upon OVA stimulation, significantly lower IL-4, IL-5, IL-10 and IL-13 levels were detected in non-responders, while IL-22 was significantly higher. Comparison of fecal microbiota revealed differences of bacterial communities on single bacterial Operational-Taxonomic-Unit level between the groups, indicating protection from food allergy being associated with a distinct microbiota composition in a non-responding phenotype in this mouse model.

## 1 Introduction

Severity and unpredictability of clinical reactions in context with food allergy are major challenges for patients, caretakers and health care personnel. The observed clinical response might differ between food allergic patients ranging from mild local symptoms like the oral allergy syndrome to severe systemic reactions such as anaphylaxis [1,2]. Actually, food allergy is among the main causes for potentially life-threatening anaphylaxis accounting for 41% of fatal reactions as reported to an European anaphylaxis registry [3]. For an efficient definition of allergy prevention measures, a profound mechanistic knowledge on sensitizing events is fundamental.

During the past years, we have investigated the association between anti-ulcer drug intake and food allergy development [4–10]. In firrst human studies in adult patients, a 3 months treatment with anti-ulcer drugs led to an increase of pre-existing food-specific IgE titers in 10% of patients, and to de novo sensitization against common dietary compounds in 15% of patients [8]. Among them, in 60% of patients with hazelnut-specific IgE clinically relevant food allergy was diagnosed by double-blind placebo controlled food challenges [10]. Further studies indicated an influence of either maternal gastric acid-suppression during pregnancy or anti-ulcer drug treatment of pediatric patients on the development of food allergy also in children [7,11–14].

Based on these murine and human data, a mouse model of oral sensitization under concomitant acid-suppression was developed being associated with induction of allergen-specific IgE, elevated Th2 cytokines and positive skin tests [5]. This immunization protocol induced severe clinical responses evidenced by positive mucosal testing, a drop of body temperature after provocations and a sustained mediator release [5,6,9].

However, in both, human and experimental studies, a certain percentage of individuals is protected from food allergy development during intake of anti-ulcer medication. This heterogeneity of reactivity especially in experimental studies with inbred mouse strains has been a matter of debate. To gain novel mechanistic insights, the overall aim of the current study was to phenotype those mice being protected from food allergy development (non-responders) in comparison with animals revealing marked systemic food allergic symptoms after immunizations based on our experimental food allergy protocol.

## 2 Material and methods

### 2.1 Animals and immunization regimen

**Fig. 1:**
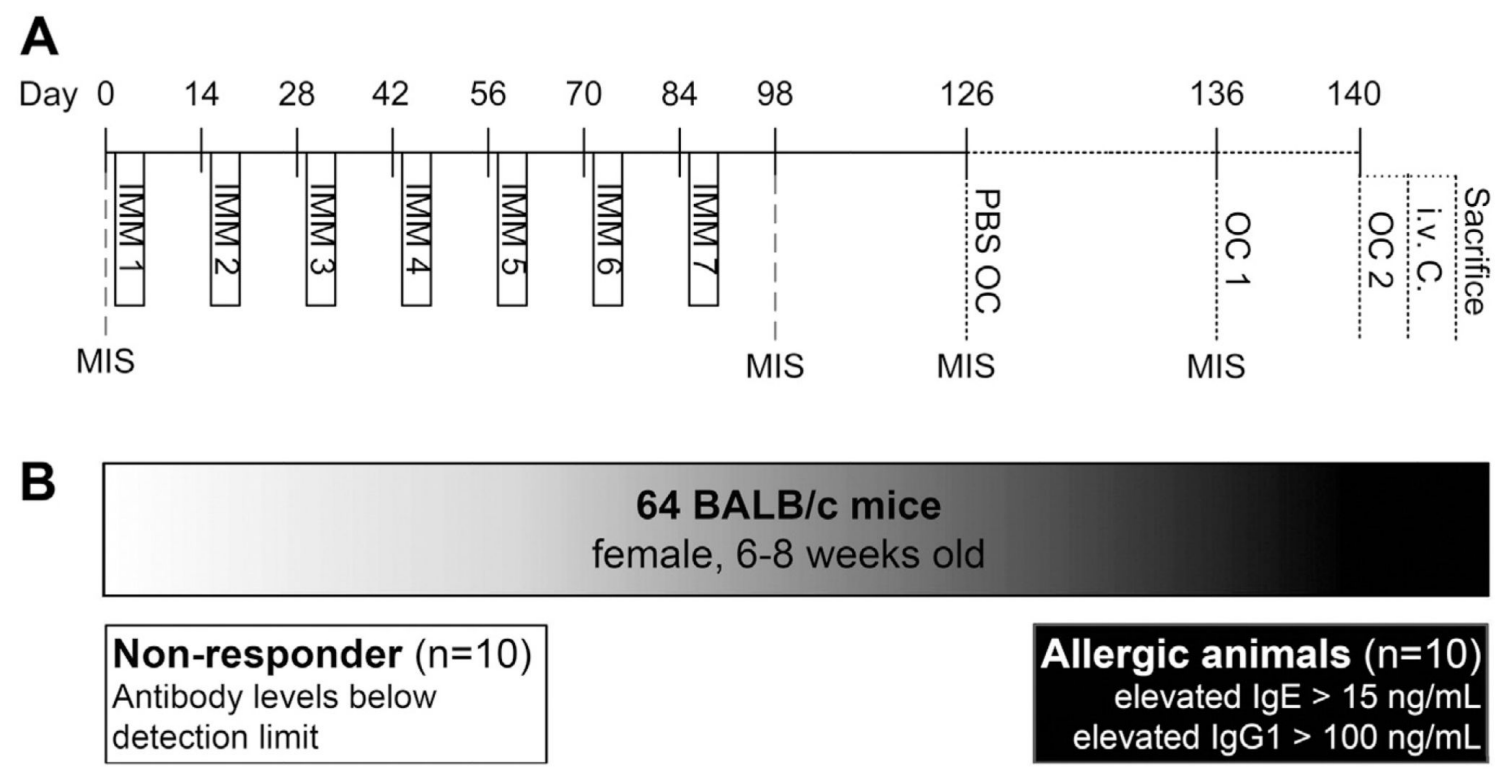
Immunization scheme and selection of animals. Sixty-four BALB/c mice were immunized (panel A) according to the protocol described in the methods section. Based on the lack of OVA-specific IgE and IgG1 antibodies, 10 animals were selected as the group of interest (non-responder, group N; panel B). They were compared to highly sensitized animals, which were characterized as being anaphylactic during the study evaluations (group A). IMM, immunization; MIS, mouse immune serum; OC, oral OVA challenge; i.v. C., intravenous challenge.

Sixty-four female BALB/cAnNCrl mice (aged 6–8 weeks, 15–20 g) were purchased from Charles River Laboratory (Charles River Laboratory, Sulzfeld, Germany). Mice were kept in polycarbonate Makrolon cages (Ehret GmbH, Emmendingen, Germany) with filter tops and espen wood bedding (Ehret GmbH, Emmendingen, Germany) and housed under conventional conditions (12 h light/dark cycle at 22 °C). The animals were kept on an ovalbumin (OVA) free diet (Ssniff, Soest, Germany) with ad libitum access to food and water. Treatment of the animals was performed by trained staff in the morning in an animal experimentation room. Animals were treated according to European Union guidelines of animal care and with permission of the ethical board of the Medical University of Vienna and the Austrian Federal Ministry of Science and Research (permission number GZ BMWF-66.009/0051-II/10b/2008). All animals were subjected to our previously established food allergy protocol [5] with modification. On days 1 to 3, animals were treated intravenously (i.v.) with the proton pump inhibitor (PPI; Losec® Astra Zeneca GmbH, Wedel, Germany; 116 μg omeprazole in 100 μL sterile sodium chloride) 2 times within 1 h. On days 2 and 3, mice were fed 0.2 mg OVA (Sigma Aldrich, Vienna, Austria, #A5503) in combination with sucralfate (2 mg; Ulcogant®, Merck, Vienna, Austria) 15 min after the second PPI i.v. injection. This immunization cycle was repeated for 7 times (Fig. 1A). Out of the total of 64 animals undergoing the immunization protocol, we defined 10 animals of interest based on their IgE and IgG1 antibody titers after the last immunization step. These ten mice revealed antibody levels below the detection limit and were classified as antibody non-responder group (group N, n = 10/64; Fig. 1B). They were compared to 10 control animals with an OVA-specific IgE antibody response above 15 ng/mL classified as highly sensitized (allergic) group (group A; n = 10/64). This cut-off level was chosen based on our numerous previous immunization studies investigating clinical response upon oral immunizations under gastric acid suppression [5,6,9] and own unpublished data. All other sensitized animals with IgE responses below 15 ng/mL and above background values as well as OVA-specific IgG1 responses (n = 44) were excluded from this study. Four weeks after the last immunization, mice were subjected to an oral PBS challenge for control purposes to exclude unspecific changes during provocation and 10 days later to an oral OVA provocation (50 mg per mouse; oral challenge 1 (OC1)). Mice were fasted overnight before oral challenges with access to water only. One hour after each challenge, blood was collected for measurements of mouse mast cell protease-1 (mMCP-1) as well as OVA uptake. Four days thereafter, animals were re-challenged with OVA i.g. (OC2) to induce a strong local intestinal allergic response. One hour later, mice were challenged i.v. (50 μg OVA in 50 μL 0.9% sodium chloride) to trigger a systemic anaphylactic response. Mice were sacrificed 15 min thereafter.

Blood samples were taken prior to the first immunization step and 2 weeks after the last immunization, 1 h after the PBS challenge as well as after the first OVA challenge (OC1).

### 2.2 Antibody measurements

Mouse sera were collected before the first and 2 weeks after the last immunization step and screened for OVA-specific IgE, IgG1, IgG2a and IgA in ELISA, as described recently [5] using rat anti-mouse IgG1, IgG2a, IgA and IgE (0.1 μg per well, BD Biosciences, Heidelberg, Germany) and peroxidase-labeled goat anti-rat IgG (1:1000, Amersham, Buckinghamshire, UK). After sacrifice, mouse intestines were removed and flushed with 2 mL extraction buffer (Complete Mini, Roche) for detection of mucosal total and OVA-specific IgA levels. For total IgA determination, microtiter plates were coated with a rat anti-mouse IgA (0.1 μg per well; BD Biosciences) overnight at 4 °C. After washing, wells were blocked with 1% bovine serum albumin in TBS containing 0.05% Tween for 2 h. Thereafter, standard dilution series or mucosal lavage fluid (diluted 1:1000) were added for 30 min. After repeated washing, a biotin-labeled anti-mouse IgA antibody (0.1 μg per well; BD Biosciences) was added for 30 min. After washing, wells were incubated with horseradish peroxidase-labeled streptavidin (1:5000, Pierce, Rockford, USA) and the color reaction was developed using tetramethylbenzidine (TMB) substrate and measured at 450 nm with reference 630 nm.

OVA-specific IgA was determined in intestinal lavage fluid as described above for serum OVA-specific IgA, except that mucosal lavage samples were applied undiluted. Antibody titers were calculated according to standard dilution series using mouse IgA, IgE, IgG1 and IgG2a antibodies (BD Biosciences) after subtraction of antibody levels before the first immunization as described before [5].

### 2.3 Systemic OVA uptake

For measurements of OVA levels in serum samples collected after OC1, microtiter plates were coated with a mouse anti-OVA capture antibody (0.1 μg per well; AbD Serotec) overnight at 4 °C. After washing, wells were blocked with 1% dry milk powder in TBS containing 0.05% Tween for 2 h. Thereafter, serum samples (diluted 1:4) were added overnight at 4 °C. After repeated washing, a rabbit anti-OVA antibody (0.025 μg per well; Thermo Scientific) was added for 2 h. After washing, wells were incubated with horseradish peroxidase-labeled anti-rabbit antibody (1:6000, Thermo Scientific) and the color reaction was developed using TMB substrate and measured at 450 nm with reference 630 nm.

### 2.4 Gastric pH measurements

The efficacy of PPI injections to elevate the gastric pH was evaluated 1 h after i.v. PPI application, as described previously [5]. The pH was measured on a pH-meter after diluting 150 μL gastric fluids in 1.3 mL distilled water. As controls, 150 μL 0.9% sodium chloride or 150 μL pH calibrating solution in distilled water were used.

### 2.5 Anaphylaxis read-out

To evaluate allergen-specific severe clinical responses after OVA challenges, mouse sera were screened for the mast cell degranulation marker mMCP-1 using the mouse mMCP-1 ELISA kit (eBioscience, Vienna, Austria, #88-7503), as described recently [8]. Serum samples were taken 1 h after PBS challenge (as negative control) and after the first oral OVA challenge (OC1). Hypothermia as a consequence of systemic anaphylaxis was assessed by measurements of rectal body temperature before and 5 and 10 min after i.v. OVA challenge.

### 2.6 Spleen cell stimulation and cytokine measurement

After sacrifice, spleens were removed under sterile conditions and spleen cells were prepared as described [5]. Spleen cells were stained for CD4^+^ CD25^+^ Foxp3^+^ T-regulatory cells with the mouse regulatory T-cell staining kit (eBioscience, #88-8111), according to the manufacturer's instructions. Absolute numbers of CD4^+^ T-cells and CD4^+^ CD25^+^ Foxp3^+^ T-cells were calculated per spleen.

For cytokine measurements, spleen cells were stimulated with OVA (0.2 μg per well), medium for 72 h. Undiluted spleen cell supernatants were screened for cytokine production using the mouse Th1/Th2/Th17/Th22 13plex FlowCytomix Multiplex kit (eBiosciences, #BMS822FF), following manufacturer's instructions. Acquisition was performed on a FACS Calibur flow cytometer (BD Biosciences) and data were analyzed using the eBioscience FlowCytomix Pro Software.

### 2.7 Histological evaluations of gastro-intestinal tissue sections

Stomach and intestine were removed under sterile conditions and put into 4% paraformaldehyde overnight and then transferred into PBS. The stomach was cut open along the sagittal plane. The intestine was transversally cut using a random start and a cutting interval of 2 cm to obtain systematic uniform random samples. Sections of paraffin embedded samples (3–4 μm thickness) were stained with haematoxylin/eosin (HE) for inflammatory infiltrates, periodic acid-Schiff reagent (PAS) for goblet cells, and chloracetate-esterase (CAE) for detection of myeloid cells in the mucosa as previously described [15].

### 2.8 Bacterial community composition in feces samples

Ten days before sacrifice, feces samples were collected from individual animals by placing the mouse into a restrainer to avoid cross-contamination. Feces samples were immediately shock-frozen in liquid nitrogen and stored at −80 °C until further processing.

About 35 mg of fecal samples per mouse were used for microbiome analyses. Total bacterial genomic DNA was extracted using NucleoSpin Kit for Soil (Macherey-Nagel, Dueren, Germany) following the manufacturer's instructions. Amplification of the V6–V9 region of 16S rRNA gene was performed with primer 926F (5′-AAACTYAAAKGAATTGACGG-3′) [16] and 630R (5′-CAKAAAGGAGGTGATCC-3′) [17] with the attached Roche 454 sequencing adaptors. For multiplexing purposes the forward primer included a 10-nt barcode sequence. Three independent PCRs were performed for each sample with Fast Start High Fidelity PCR System (Roche, Mannheim, Germany) containing 20 ng of template DNA with an optimal annealing temperature of 50 °C and 22 cycles. PCR reactions were pooled and purified using QiaQuick PCR Purification Kit (Qiagen, Hilden, Germany). After quantification using a Quant-iT™ PicoGreen dsDNA quantification kit (Invitrogen, Paisley, UK), samples were equally pooled. The sequencing of this amplicon library was performed on a Roche 454 GS FLX Pyrosequencer (Roche, Mannheim, Germany) using Titanium chemistry. Amplicons were sequenced unidirectionally as recommended in the manufacturer's instruction for amplicon Lib-L libraries. Sequences were processed and data were analyzed according to the 454 Schloss standard operating procedure (SOP; http://www.mothur.org/wiki/Schloss_SOP) [18] with the software Mothur v.1.29.0 [19]. Reads were denoised, quality filtered and trimmed. For taxonomic analysis, sequences were aligned against Silva SEED alignment database [20], chimeras were removed using UCHIME implementation [21] in Mothur, and taxonomic assignment was performed using RDP trainset with a cut-off of 80% [22]. To compare equal numbers of sequences of each fecal sample, subsamples with 10,429 sequences were generated. Sequences with similarity >97% were combined to one OTU. Prior to the statistical analysis, all OTUs with <0.01% of the total abundance were excluded from the analysis.

### 2.9 Statistical analysis

Data evaluation was done using GraphPad Prism 5 software. First, results were tested for normal distribution followed by unpaired *t*-test. Cytokine levels results were analyzed using two-way ANOVA and Bonferroni multiple comparison test. A p-value < 0.05 was considered statistically significant.

Statistical analysis of gut microbiome data was performed using R platform (R version 2.15.1) with the packages VEGAN [23] and ade4 [24] and custom R scripts. Hellinger transformed OTU abundances were used to compare community patterns between the two groups by Principal Component Analysis (PCA). In addition, a multivariate analysis of variance (npMANOVA), based on Bray-Curtis distance on relative OTU abundances was performed. Wilcoxon-Mann Whitney tests were performed on OTU levels to compare groups N and A. Differences with an p-value < 0.01 were considered to be statistically significant.

## 3 Theory

Using an inbred mouse strain with all animals housed under identical conditions and immunized following a standardized protocol, a uniform immune response would be expected. Due to different responses in previous experiments we aimed to retrospectively phenotype animals, which were protected from food allergy development, and compare them to allergic control animals. This work should establish a sound basis for further studies to prospectively induce a phenotype protected from food allergy development, with the final aim of developing allergy prevention strategies.

## 4 Results

### 4.1 Determination of specific antibody levels allows classification of mice as non-responders versus highly sensitized animals

**Fig. 2:**
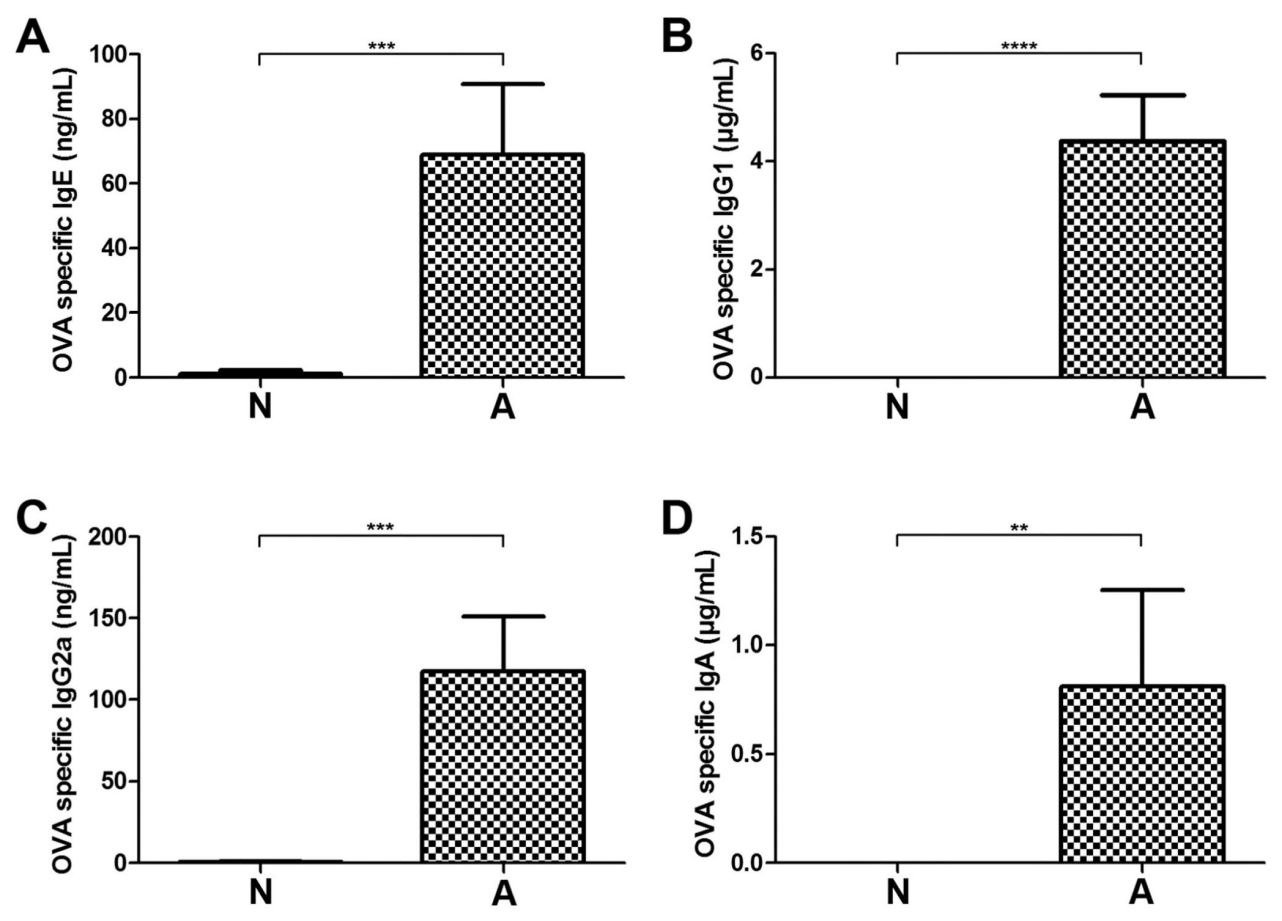
OVA-specific antibody profile in murine sera. OVA-specific IgE (A), IgG1 (B), IgG2a (C) and IgA (D) levels were determined by ELISA. According to the serological status, animals of interest were selected as antibody non-responders (group N) and compared to highly sensitized control animals (group A). Data represent mean + standard error of the mean (SEM); ****p < 0.0001, ***p < 0.001, **p < 0.01.

Sixty-four mice were subjected to our protocol of oral food allergy induction (Fig. 1A). After the last immunization step, 10 out of 64 animals of interest were identified based on lack of OVA-specific antibodies (Fig. 1B), i.e. non-responder animals, group N (Fig. 2A–D). They were compared to 10 control mice (10/64), which mounted high levels of OVA-specific IgE (Fig. 2A), IgG1 (Fig. 2B), IgG2a (Fig. 2C) and IgA (Fig. 2D) responses. These animals were classified as the highly sensitized, allergic group A. Mice with OVA-specific IgE below 15 ng/mL but above background levels, as well as with specific IgG1 responses were excluded from further analysis (n = 44).

**Fig. 3:**
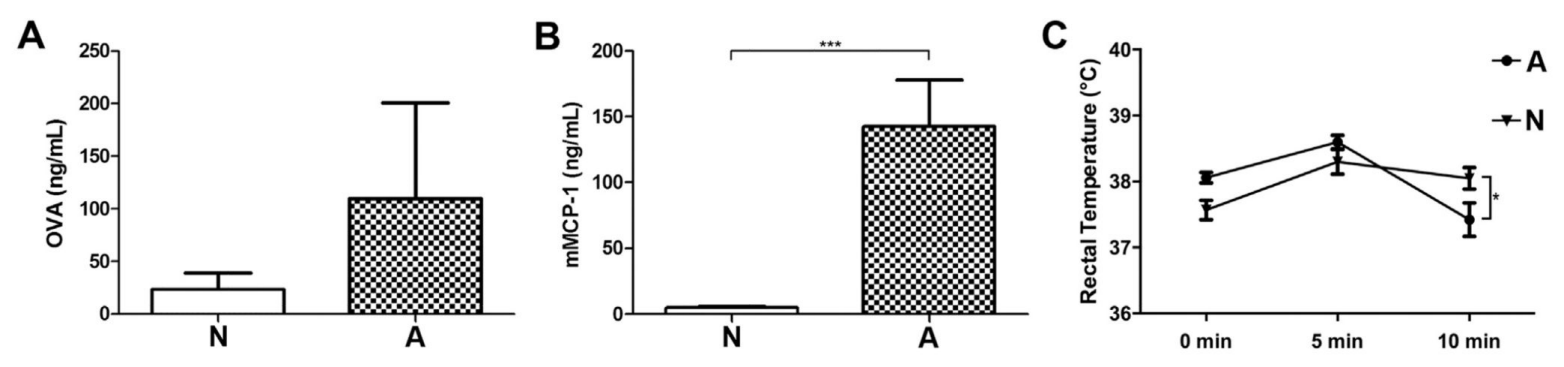
Differences in antigen uptake and clinical responses. After OVA gavage, higher OVA concentrations in serum (A) were found in allergic mice (group A). Mouse MCP-1 levels (B) were significantly elevated in group A after the first oral OVA challenge (OC 1). Rectal temperature (C) was measured before and 5 and 10 min after i.v. OVA provocation of antibody non-responders (triangle) and highly sensitized animals (circles). Data represent mean + SEM; *p < 0.05.

For read-out of clinical symptoms animals were orally challenged with OVA twice to induce a strong mucosal response. Systemic OVA uptake was evaluated in serum samples after oral challenges (Fig. 3A). We observed higher levels of circulating OVA in the highly sensitized animals (group A), which might point towards an enhanced intestinal uptake compared to non-responder animals (group N). Additionally, mice were subjected to an i.v. OVA provocation to investigate their potential for a systemic anaphylactic response. Mouse mast cell protease-1 (mMCP-1) levels, which indicate a mast cell-dependent anaphylactic response, were measured in serum taken 1 h after PBS and first oral OVA challenge (OC1) (Fig. 3B). We detected only baseline mMCP-1 levels in challenged non-responders (group N), which were significantly lower as compared to the allergic mice. To ensure the antigen-specificity of the anaphylactic response, mice were also challenged with PBS 10 days before the first OVA challenge. Mouse MCP-1 titers were below 10 ng/mL and did not differ between the 2 groups after oral PBS challenges (data not shown). Additionally, 8/10 mice of the allergic animals developed a reduction of body temperature, being defined as a decrease of at least 0.5 °C, measured after oral OVA provocation (data not shown). The temperature drop 5 and 10 min after i.v. OVA challenge was significantly more pronounced in the allergic animals of group A (Fig. 3C).

### 4.2 Serological differences are associated with distinct intestinal IgA levels

**Fig. 4:**
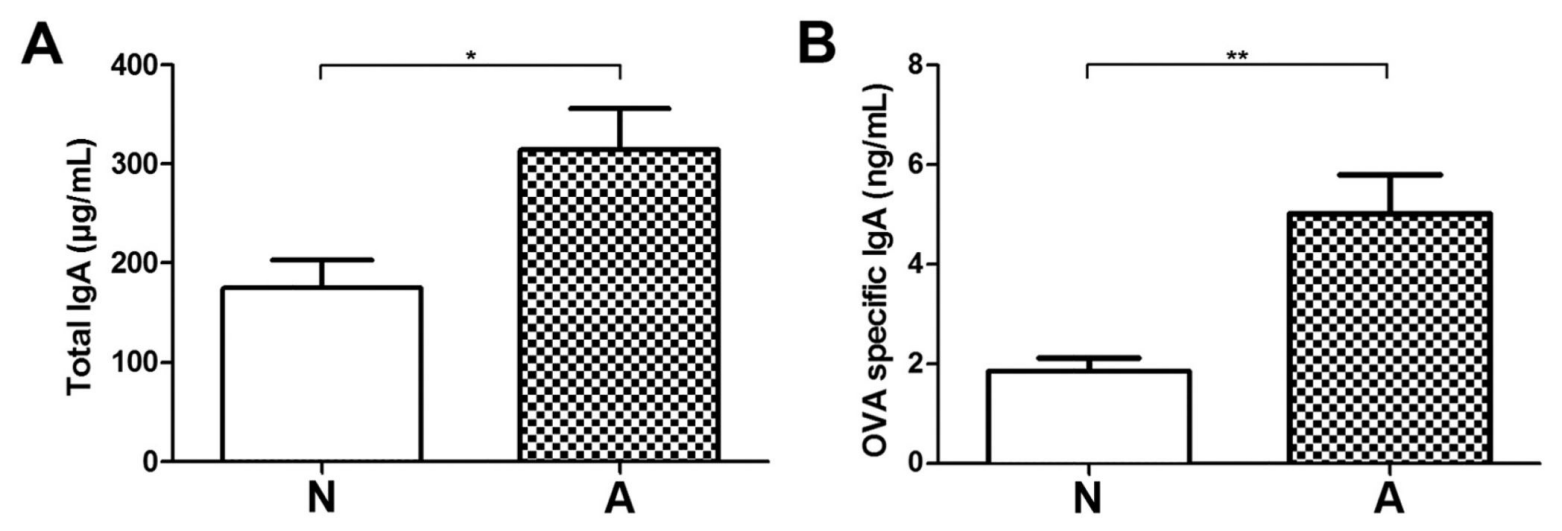
IgA levels in intestinal lavage fluid. Total (A) and OVA-specific IgA (B) were measured in intestinal lavage fluid by ELISA. Data represent means + SEM; **p < 0.01, *p < 0.05.

As a surrogate marker for mucosal humoral defense and potential protective factor in food allergic responses, total IgA and OVA-specific IgA in intestinal lavage fluids of the immunized animals were measured. Intestinal total (Fig. 4A) and OVA-specific IgA (Fig. 4B) levels were significantly lower in the non-responders (N) compared to allergic animals (A).

**Fig. 5:**
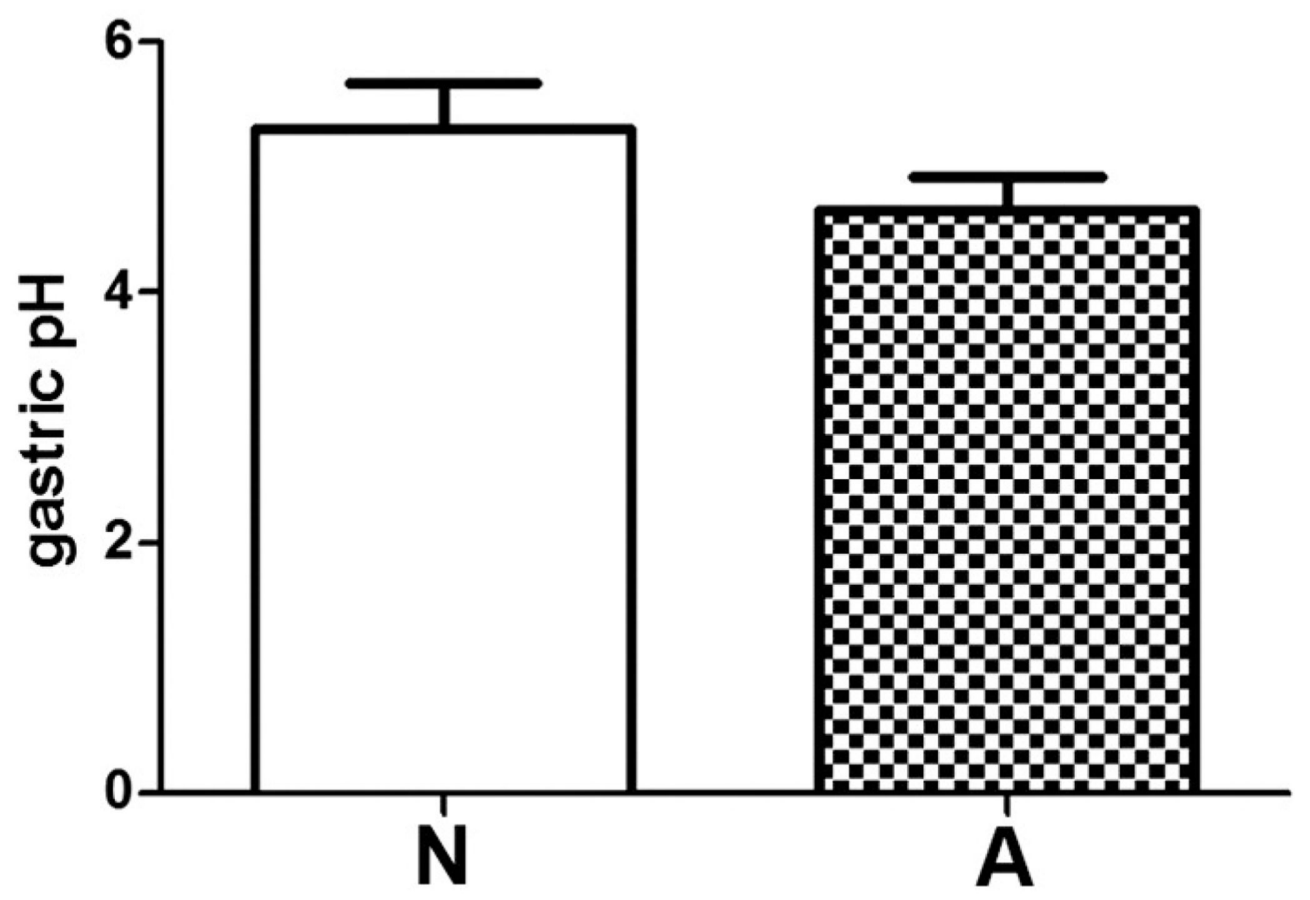
Gastric pH measurement after anti-ulcer medication. Acid-suppressive medication induces comparable elevated intragastric pH levels in all animals. Gastric pH was measured one hour after i.v. PPI injection on day of sacrifice. Data represent mean + SEM.

To rule out the possibility that the differences in antibody production might be caused by variable responsiveness of mice to the gastric acid-suppressive effect of PPIs, we performed gastric pH measurements 1 h after PPI injection. Comparable elevated gastric pH levels were determined in both groups (Fig. 5).

**Fig. 6:**
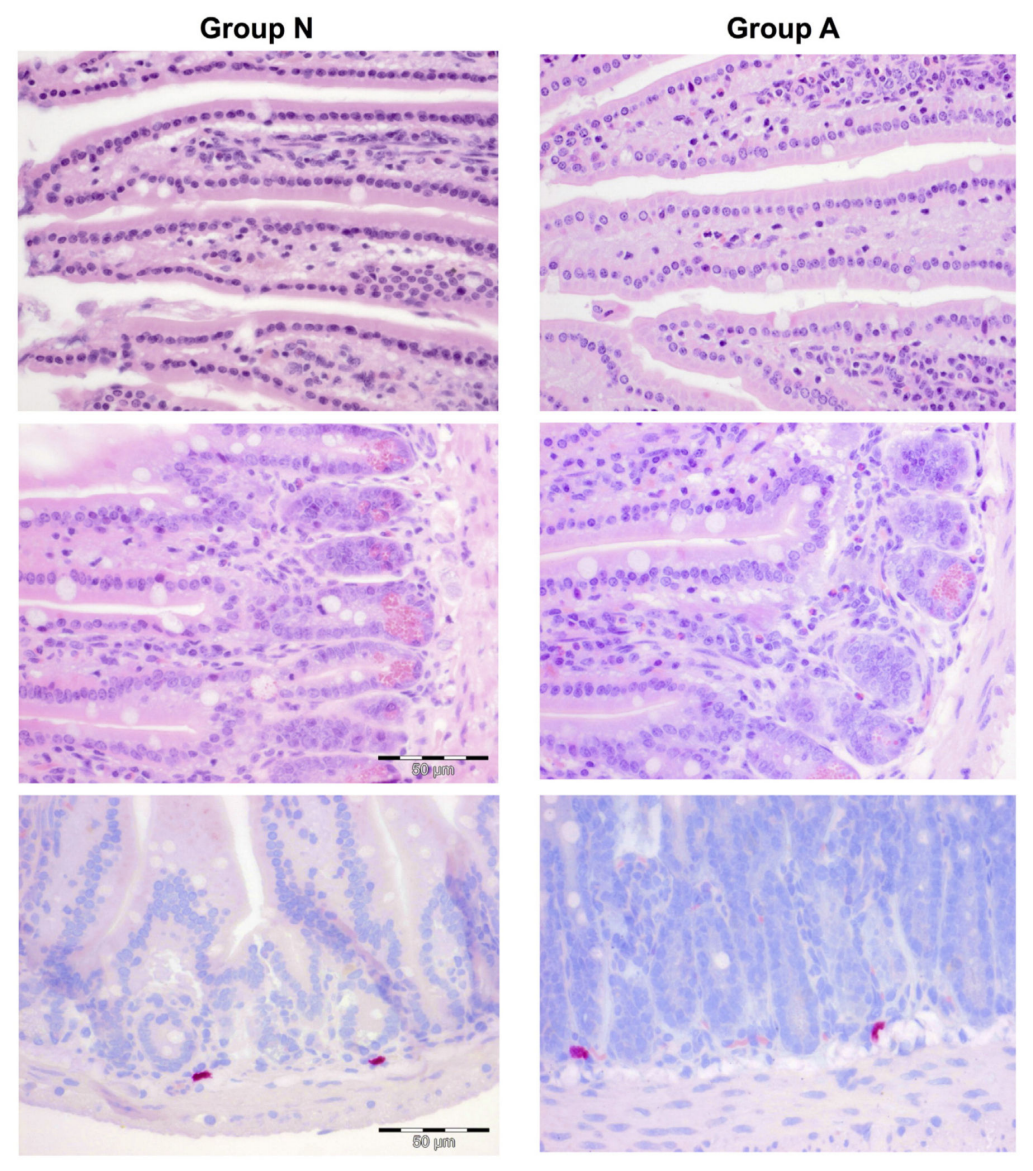
Histology of mouse intestines. Representative micrographs of paraffin sections of jejunum stained with HE (upper row: apical portion of villi, middle row: basal portion of villi; 1:40) and CAS staining (bottom row, 1:40) are shown. Left panels for group N and right panels for group A. No significant differences were found with respect to epithelium, infiltrating eosinophils and myeloid / mast cells .

Sagittal sections of the stomach and transverse, uniform systematic random sections of the intestine stained with HE and CAE for inflammatory infiltrates, and myeloid mucosal cells, respectively, showed comparable gastrointestinal morphology between the groups (Fig. 6).

### 4.3 IgE responders have stronger Th2 responses, but also higher T-regulatory cell numbers

**Fig. 7:**
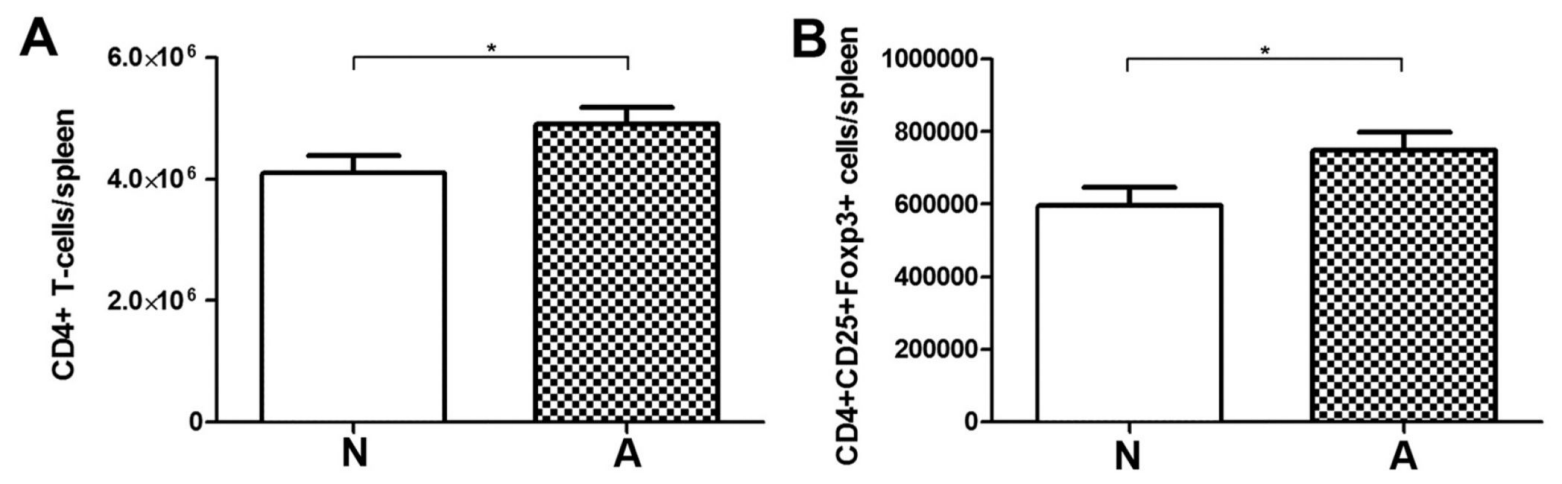
T-regulatory cell subset staining of splenocytes. Significantly lower numbers of T-cells are found in spleens of antibody non-responder animals. Absolute numbers of CD4+ T-cells (A) and T-reg cells (CD4 + CD25 + Foxp3+) (B) were evaluated by flow cytometry. Data are mean + SEM; *p < 0.05.

To assess whether differences on the antibody level were accompanied by differences in T-regulatory cell subsets, spleen cells were stained for CD4, CD25 and Foxp3 and analyzed by flow cytometry. No significant differences in total spleen cell numbers were found between the two groups. The absolute numbers of CD4^+^ T-cells (Fig. 7A) and CD4^+^ CD25^+^ Foxp3^+^ T-regulatory cells (Fig. 7B) were significantly lower in the non-responder animals (N) compared to allergic mice (A). Cytokine levels were measured in supernatants of spleen cells, which were stimulated with medium, to investigate the background levels of cytokine production, or with OVA, to induce an allergen-specific cytokine response. In a FlowCytomix multiplex approach, 13 different cytokines were measured (Table 1). In line with the increased IgE response the Th2 type cytokines IL-4, IL-5 and IL-13 were significantly higher after OVA stimulation in the allergic mice than in group N. No group differences were observed for IL-22, however, this cytokine was significantly elevated within group N when antigen-specific splenocyte stimulation was compared to stimulation with medium control. Interestingly, the T-regulatory cell associated cytokines IL-10 and IL-27 were higher in the allergic animals (group A) being in line with the higher numbers of T-reg cells, indicating a counter-regulatory mechanisms in this group. IL-2 as cytokine for T-cell proliferation and differentiation was significantly elevated in the OVA-stimulated spleen cells of allergic mice (Table 1).

### 4.4 Bacterial OTU differences in feces samples of non-responder mice and anaphylactic animals

**Fig. 8:**
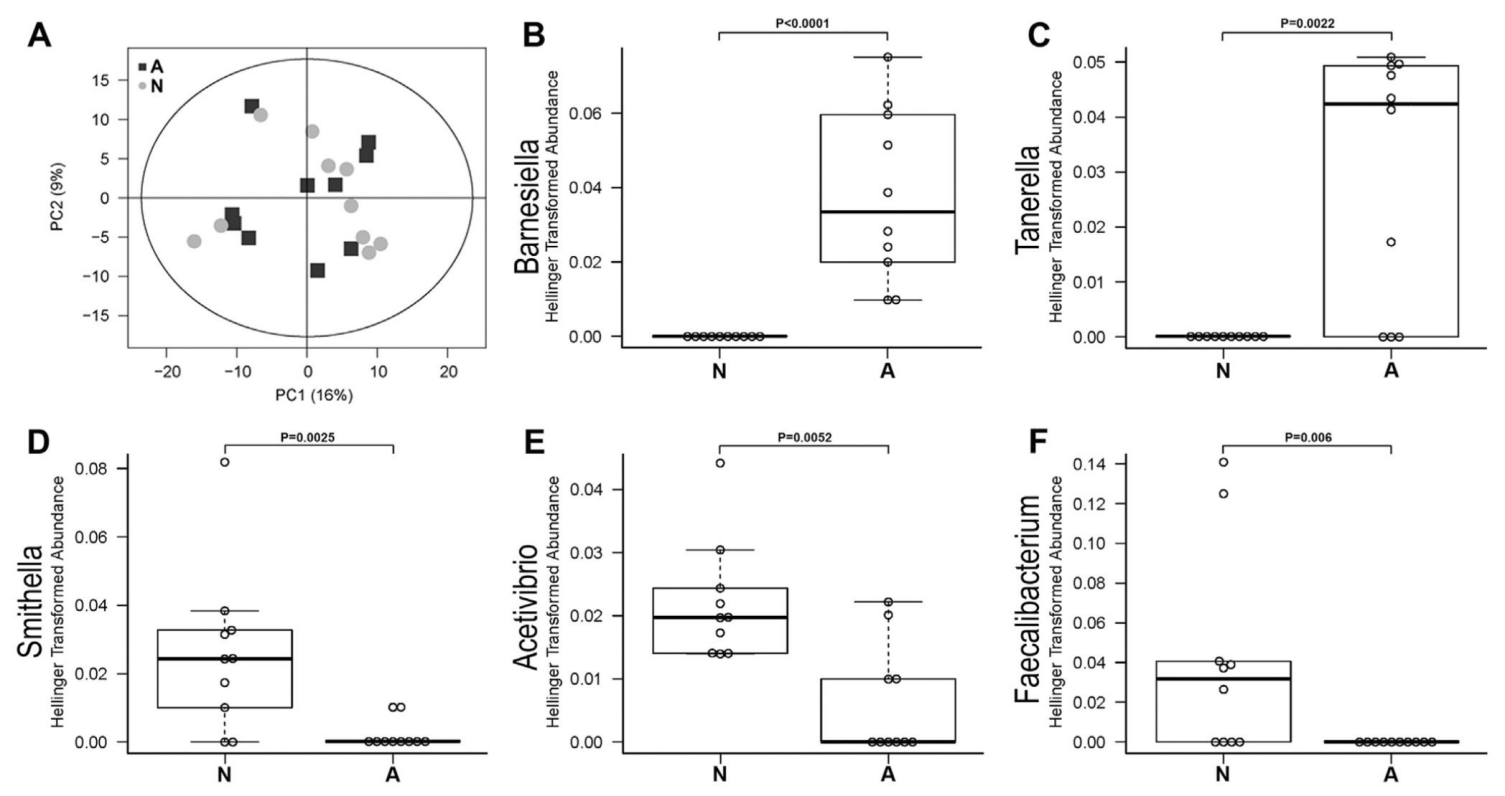
Analysis of bacterial community composition of murine feces samples of non-responder (N) and allergic (A) mouse groups. Hellinger transformed sequence abundance data on OTU levels were analyzed by principal component analysis (A). Relative abundances of *Barnesiella* (OTU 185; B), *Tannerella* (OTU 213; C), *Smithella* (OTU 233; D), *Acetivibrium* (OTU 289, E) and *Faecalibacterium* (OTU 102; F) are shown in boxplot analyses for each group N and A. The boxes represent the inner quartile value range with the median indicated as black line.

Feces samples of individual mice were analyzed to identify gut bacterial community composition. 454-pyrosequencing of the V6-V9 region of the 16S rRNA gene was performed. After quality check and subsampling, an output of 10,429 sequences per fecal sample was obtained. All sequences with at least 97% similarity were considered as 1 OTU resulting in the detection of 409 OTUs. Principal component analysis (PCA) demonstrated only very limited clustering of OTUs identified in the fecal samples of animals within the same group (PCA; Fig. 8A). npMANOVA analysis was used to compare the group of interest with the allergic control animals for differences in the bacterial community composition on OTU level. No significant differences could be shown (p = 0.349). After taxonomic classification of sequence data, we did not detect OTUs of the *Porphyromonadaceae* family assigned to *Barnesiella* (OTU 185) and *Tannerella* (OTU 213) genus (Fig. 8B and C) in the non-responders. Therefore, these two OTUs belonging to the *Porphyromonadaceae* family were not present in animals being protected from food allergy. Higher abundances of sequences showing high similarities to *Synthrophaceae* (*Deltaproteobacteria*) and *Ruminococcaceae* (*Firmicutes*, *Clostridia*) were found in the antibody non-responder animals (group N) compared to the allergic animals (group A), where *Smithella* (OTU 233, Fig. 8D) and *Faecalibacterium* (OTU 102, Fig. 8F) were not found and the abundance of *Acetivibrio* (OTU 289, Fig. 8E) was significantly lower.

## 5 Discussion

Food allergy does affect patients' quality of life and represents an enormous economic burden [25] with major efforts for patients, their caretakers and regulatory authorities. Therefore, researchers have focused on identifying underlying mechanisms of food allergy development using animal models. Although substantial differences are known between human and mouse allergic responses, food allergy mouse models provide important and useful information on disease mechanisms [26,27]. However, a number of different routes and protocols of sensitizations has been published so far, rendering direct comparison of results from studies highly complex [28].

The advantage of our oral food allergy model in BALB/c mice is the physiological sensitization route applying the allergen intragastrically under acid suppressive medication without further adjuvants [5]. Even though in our animal experiments inbred mice were housed and sensitized under identical conditions, immune responses were not uniform [4–7,9,10,29–32] (and own unpublished data). Thus, in the present study we aimed to phenotype mice being protected from food allergy in comparison to mice being highly sensitized and anaphylactic after immunizations.

Based on this retrospective approach, we identified 3 distinct features of animals protected from food allergy development: 1) a tight intestinal epithelium, 2) elevated levels of IL-22 and 3) a distinct microbial composition.

The integrity of the intestinal epithelium is one of the first major regulatory mechanisms against the development of food allergy. A “leaky gut”, meaning the damage of the epithelial barrier function and an increased uptake of food antigens and allergens into the system, can facilitate allergic responses if other local pro-inflammatory signals are present. In our model we observed significantly lower serum concentrations of orally applied OVA only in the animals protected from food allergy, which might indirectly point towards a lower uptake of the allergen via the mucosa. Functional intestinal epithelial integrity has been linked to secretory immunity as mice deficient for sIgA and IgM have increased mucosal leakiness [33,34]. In general, mucosal IgA is important for the immunological defense against exogenous antigens and pathogens by exclusion, and thus, might be protective and lead to mucosal tolerance [35]. Some studies revealed tolerant mice to have more secretory IgA than sensitized animals [36]. However, also in mice deficient for the polymeric immunoglobulin receptor, and thus lacking secretory IgA, oral tolerance can be induced. This observation led to the conclusion that IgA is probably not the only control mechanism of oral tolerance [37,38]. In contrast, a recent study using a cholera toxin-based mouse model of cow's milk allergy revealed high IgA levels in plasma and colon homogenates upon a 6 weeks immunization protocol [39]. It might be hypothesized that in food allergy increased amounts of allergen-specific IgA are secreted into the intestine as counter mechanism to avoid intestinal allergen uptake or as a result of the overall enhanced immune response, which might be the explanation for the observed higher local and systemic IgA levels in allergic mice in our food allergy model.

In addition to the local secretory immune differences we observed changes in intestinal bacterial colonization patterns between the groups. Although inbred animals were housed and treated identically, non-allergic animals (group N) revealed significantly higher abundances of sequences belonging to *Synthrophaceae* (*Deltaproteobacteria*) and *Ruminococcaceae* (*Firmicutes*, *Clostridia*), whereas two OTUs of the *Porphyromonadaceae* family were more abundantly present only in allergic animals (group A). Variations in gut microbiota are associated with the induction of several diseases, including diabetes, obesity and cancer [40] and were strongly linked to the development of atopic disorders [41], including food allergy [42]. In line with the hygiene hypothesis [43], a reduced or altered microbial load may insufficiently counterbalance a Th2 response thus favouring the occurrence of food allergy [44]. In mice, it appears that even minor differences in the gut microbiome can have substantial effects on experimental models of disease. Inbred mice with the same genetic background obtained from two different suppliers featured different dominant microbial communities [45]. Changes in microbial composition were further induced by variations of diet, by moving young mice from one room to another or by stress [40,46]. Therefore, inconsistencies within animal experiments may be related to the observed differences in the bacterial composition. In our mouse model, animals were obtained from the same supplier. Offspring of different mothers were randomly grouped and were all fed the same OVA-free diet. Interestingly, *Tannerella forsythensis* and *Porphyromonas gingivalis*, both belonging to the *Porphyromonadaceae* family, which was present at significantly higher levels in allergic mice in our study, were shown to stimulate a strong pro-inflammatory epithelial immune response under anaerobic conditions [47]. With regard to food allergy, the *Porphyromonadaceae* family was also found in OVA sensitized *Il4raF709* mice together with *Rikenellaceae* species, to discriminate between OVA sensitized and PBS sensitized mice [48].

Moreover, *Ruminococcaceae*, belonging to the system of Clostridiales, might be linked to IL-22, as Clostridia-containing microbiota protected against sensitization to food allergens and induced IL-22 [49], which has been characterized for its protective and inflammatory functions and its regulatory role on intestinal epithelial integrity [50,51]. In line with these results we observed higher levels of IL22 after OVA stimulation of spleen cells in tolerant mice, which had higher abundances of *Ruminococcaceae*, compared to allergic animals. However, at this point it can only be speculated whether the OTUs more abundantly present in the tolerant group can explain the non-responsiveness of these animals. As we collected feces samples only shortly before sacrifice, it is not possible to draw conclusions on bacterial composition before and during food allergy development. We do not know at this point whether the significantly more abundant OTUs were found in the respective animals already before sensitization and, therefore, had a direct influence on the development of the immune response, or whether the suppression of these OTUs occurred as a result of the immunization process and had an indirect effect on the sensitization.

In conclusion, our food allergy animal model likely reflects the situation in human patients. By phenotyping animals being protected from food allergy we determined a reduced uptake of OVA, presumably due to a tighter epithelium, elevated allergen-specific IL-22 levels and absence or higher abundance of distinct bacterial strains to be associated with allergy protection. Without any doubt, it will be essential for future studies to evaluate whether changes of microbiota composition as observed in this retrospective evaluation can prospectively achieve induction of a phenotype protected from food allergy development.

## Figures and Tables

**Table 1:** Cytokine profiling of unstimulated and OVA stimulated spleen cells.

	N						A									
	Medium			OVA			Medium			OVA			P (N)	P (A)	P (N-A)	P (N-A)
	Mean	SEM	95% CI	Mean	SEM	95% CI	Mean	SEM	95% CI	Mean	SEM	95% CI	Med-OVA	Med-OVA	Med	OVA
IL 13	0	0	(0–0)	0	0	(0–0)	104.20	(74.49)	(−64.30−272.70)	445.70	(199.80)	(−6.18−897.60)	n.s.	0.024	n.s.	0.011
IL 4	4.35	(4.35)	(−5.49−14.20)	6.50	(4.09)	(−2.76−15.75)	55.64	(30.83)	(−14.11−125.40)	136.50	(63.30)	(−6.70−279.70)	n.s.	0.021	n.s.	0.027
IL 5	11.13	(7.48)	(−6.63−28.88)	23.08	(12.31)	(−4.76−50.92)	26.93	(12.33)	(−0.97−54.83)	83.28	(26.80)	(22.66−143.90)	n.s.	0.014	n.s.	0.028
IL 22	1.93	(1.93)	(−2.44−6.31)	17.60	(5.27)	(5.69−29.51)	6.52	(3.76)	(−1.98−15.01)	16.47	(5.47)	(4.08−28.85)	0.012	n.s.	n.s.	n.s.
IL 17	0	(0)	(0–0)	4.51	(3.07)	(−2.43−11.44)	16.01	(12.42)	(−12.08−44.10)	37.48	(30.68)	(−31.93−106.90)	n.s.	n.s.	n.s.	n.s.
IL 10	0	(0)	(0–0)	0	(0)	(0–0)	0	(0)	(0–0)	25.18	(14.62)	(−7.89−58.25)	n.s.	n.s.	n.s.	0.040
IFN-γ	3.86	(3.29)	(−3.57−11.30)	27.85	(13.10)	(−1.79−57.48)	215.80	(120.50)	(−56.89−488.40)	477.00	(319.10)	(−244.90−1199)	n.s.	n.s.	n.s.	n.s.
IL 1a	0	(0)	(0–0)	3.80	(3.30)	(−3.66−11.27)	3.70	(3.70)	(−4.67−12.06)	0	(0)	(0–0)	n.s.	n.s.	n.s.	n.s.
TNF-α	0	(0)	(0–0)	6.75	(4.09)	(−2.50−16.01)	0	(0)	(0–0)	0	(0)	(0–0)	n.s.	n.s.	n.s.	n.s.
IL 27	0	(0)	(0–0)	0	(0)	(0–0)	21.49	(7.30)	(4.97–38.00)	3.57	(2.38)	(−1.82−8.96)	n.s.	0.003	0.001	n.s.
IL 6	9.13	(5.91)	(−4.24−22.50)	17.34	(5.24)	(5.47–29.20)	10.99	(4.20)	(1.48–20.50)	36.02	(9.88)	(13.67–58.36)	n.s.	0.017	n.s.	n.s.
IL 21	0	(0)	(0–0)	4.55	(4.55)	(−5.75−14.85)	0	(0)	(0–0)	0	(0)	(0–0)	n.s.	n.s.	n.s.	n.s.
IL 2	40.15	(12.11)	(12.76–67.54)	57.10	(16.99)	(18.66−95.54)	63.30	(17.55)	(23.61–103.00)	128.7	(19.51)	(84.51–172.80)	n.s.	0.003	n.s.	0.009

Abbreviations: SEM: standard error of mean, CI: confidence interval, Med: medium, n.s.: not significant.

## Abbreviations

OVA: ovalbumin
i.v.: intravenous
PPI: protonpump inhibitor
OC: oral challenge
mMCP-1: mouse mast cell protease-1
i.g.: intragastric
TMB: tetramethylbenzidine
HE: haematoxylin/eosin
PAS: periodic acid-Schiff reagent
CAE: chloracetate-esterase
OTU: operational taxonomic unit
